# Elevated central venous pressure is associated with impairment of microcirculatory blood flow in sepsis: a hypothesis generating post hoc analysis

**DOI:** 10.1186/1471-2253-13-17

**Published:** 2013-08-07

**Authors:** Namkje AR Vellinga, Can Ince, E Christiaan Boerma

**Affiliations:** 1 Department of Intensive Care Adults, Erasmus MC University Medical Center, Room H625, P.O. Box 2040, Rotterdam 3000 CA, Netherlands; 2 Department of Intensive Care, Medical Center Leeuwarden, P.O. Box 888, Leeuwarden 8901 BR, Netherlands; 3 Department of Translational Physiology, Academic Medical Center, P.O. Box 22660, Amsterdam 1100 DD, Netherlands

**Keywords:** Microcirculation, Sepsis, Central venous pressure, Sidestream dark field imaging

## Abstract

**Background:**

Microcirculatory driving pressure is defined as the difference between post-arteriolar and venular pressure. In previous research, an absence of correlation between mean arterial blood pressure (MAP) and microcirculatory perfusion has been observed. However, the microcirculation may be considered as a low pressure compartment with capillary pressure closer to venous than to arterial pressure. From this perspective, it is conceivable that central venous pressure (CVP) plays a more important role in determination of capillary perfusion. We aimed to explore associations between CVP and microcirculatory perfusion.

**Methods:**

We performed a post-hoc analysis of a prospective study in septic patients who were resuscitated according a strict non-CVP guided treatment protocol. Simultaneous measurements of hemodynamics and sublingual Sidestream Dark Field imaging were obtained 0 and 30 minutes after fulfillment of resuscitation goals. Data were examined for differences in microcirculatory variables for CVP ≤ or > 12 mmHg and its evolution over time, as well as for predictors of a microvascular flow index (MFI) < 2.6.

**Results:**

In 70 patients with a mean APACHE II score of 21, 140 simultaneous measurements of CVP and sublingual microcirculation (vessels < 20 µmeter) were obtained. (MFI) and the percentage of perfused small vessels (PPV) were significantly lower in the ‘high’ CVP (> 12 mmHg) group as compared to patients in the ‘low’ CVP (≤12 mmHg) group (1.4 ± 0.9 vs. 1.9 ± 0.9, P = 0.006; and 88 ± 21% vs. 95 ± 8%, P = 0.006 respectively). Perfusion pressure (MAP–CVP) and cardiac output did not differ significantly between both CVP groups. From time point 0 to 30 minutes, a significant increase in MFI (from 1.6 ± 0.6 to 1.8 ± 0.9, P = 0.027) but not in PPV, was observed, while CVP and perfusion pressure significantly decreased in the same period. In a multivariate model CVP > 12 mmHg was the only significant predictor for a capillary MFI < 2.6 (Odds ratio 2.5 (95% confidence interval 1.1-5.8), P = 0.026).

**Conclusion:**

We observed a significant association between a higher CVP and impairment of microcirculatory blood flow. Further research is needed to elaborate on our hypothesis generating findings that an elevated CVP may act as an outflow obstruction of organ perfusion.

## Background

Resuscitation of critically ill patients is based on the generally accepted paradigm that cardiovascular optimization is needed to ensure adequate oxygen delivery to the tissue. However, whether corrections of systemic hemodynamics are translated into improvement of oxygen delivery at the capillary level often remains unclear in the clinical setting. Indeed, the common finding in studies using microcirculatory monitoring is the absence of a clear association between microcirculatory and macrohemodynamic variables, especially in conditions of sepsis [1]. This appears to be clinically relevant: persisting sublingual microcirculatory alterations after macrohemodynamic optimization are known to be associated with adverse outcome [2]. Attempts to improve microcirculatory perfusion by increasing mean arterial pressure (MAP) has shown to be beneficial in some patients, whereas in some patients no effect or a worsening of microvascular perfusion has been observed [3-6]. The absence of a clear association between microcirculation and macrocirculation may in part be explained by the clinical setting. Since clinicians are trained to keep systemic hemodynamic variables within a certain range, extreme values are less likely to be present in clinical datasets and might therefore cloud the presence of associations between the two vascular compartments. An alternative explanation may be hidden in fundamental physiological theory. Maintenance of arterial blood pressure within the range of autoregulation is generally accepted as the main prerequisite for organ perfusion. In this paradigm the central venous pressure (CVP) is only relevant as a relatively small determinant in the net driving pressure, defined as MAP minus CVP. However, from the perspective of the microcirculation, the steep part of the pressure drop occurs upstream at the level of small arterioles (resistance vessels). The microcirculation itself may be considered as a very low pressure compartment. Therefore, mean capillary pressure is much closer to venous than to arterial pressure. From this perspective, CVP now becomes a major determinant of capillary blood flow. Because microcirculatory driving pressure is the net result of post arteriolar minus venular pressure, one might postulate that even a relatively mild increase in CVP may considerably influence the capillary perfusion pressure [3,7,8]. This issue invites a study in search of a possible association between raised CVP and microcirculatory flow. To this end we analyzed combined data of Sidestream Dark Field (SDF) in-vivo microscopy and CVP in septic patients. We hypothesized there would be impairment of microvascular blood flow under conditions of elevated CVP.

## Methods

### Patients and protocol

This study is a post-hoc analysis of a single center prospective study of the microcirculation in patients ≥ 18 years of age with confirmed sepsis, included within 4 hours of intensive care unit (ICU) admission [9]. Patients were resuscitated according a strict non-CVP guided treatment protocol aiming for the following resuscitation goals: a MAP of ≥ 60 mmHg, a mixed venous oxygen saturation (SvO_2_) of ≥ 70% and a cardiac index (CI) ≥ 2.5 L/min/m^2^. Systemic hemodynamic assessment was achieved through continuous invasive monitoring of arterial blood pressure and continuous cardiac output and SvO_2_ measurements using a pulmonary artery catheter (Vigilance, Edwards Lifesciences, Saint-Prex, Switzerland). Stepwise goal-directed protocolized resuscitation consisted of 1) repeated infusions of at least 250 mL of crystalloids, colloids, or blood products, until the increase in left ventricular stroke volume was < 10%. An upper limit of pulmonary artery wedge pressure of 18 mmHg was used as an extra precaution to avoid fluid overload.; 2) treatment of inadequate systemic oxygen supply (defined as CI < 2.5 L/min/m^2^ or SvO_2_ < 70%) with dopamine administered at up to 10 μg/kg per minute and additional enoximone in case of an inadequate response to dopamine; and 3) treatment with norepinephrine in case of MAP < 60 mmHg despite the aforementioned steps. For this post-hoc analysis, macro- and microhemodynamic data obtained directly after fulfillment of resuscitation endpoints (T0) as well as 30 minutes thereafter (T30) were analyzed. During the study period, therapeutic goals and resuscitation protocol remained unchanged. The study was approved by the local ethics committee (Medical Research Ethics Committee, Medical Center Leeuwarden, the Netherlands). Written informed consent was obtained from all included patients or their legal representative in accordance with local legislation.

### SDF imaging and analysis

A SDF camera (MicroScan, MicroVision Medical, Amsterdam, the Netherlands) is a form of handheld intravital microscopy enabling direct visualization of the microcirculation [10]. In short, the SDF camera emits stroboscopic green light with a wavelength (530 nm) within the absorption spectrum of hemoglobin, thereby depicting erythrocytes as black cells on the screen. The area of visualization is 1 mm^2^. Offline software-assisted analysis (AVA 3.0, MicroVision Medical, Amsterdam, the Netherlands) yields information on both red blood cell velocity (convective oxygen transport) and capillary density (diffusion distance): the semi-quantitative microvascular flow index (MFI), ranging from 0 (no flow) to 3 (continuous flow), and percentage of perfused vessels (PPV) provide information on convexity, whereas total vessel density (TVD) and perfused vessel density (PVD) provide information on diffusion [11]. SDF imaging as well as subsequent image analysis were performed in line with international consensus [12,13].

### Data collection and statistical analysis

Data on SDF imaging, hemodynamic variables, inotrope dose and lactate, collected at 0 (T0) and 30 minutes (T30) after fulfillment of resuscitation goals, were analyzed to examine the relationship between CVP and sublingual microcirculation. Microcirculatory variables were restricted to small vessels (< 20 μm) only. Statistical analysis was performed using SPSS 18 (IBM, New York, USA). Differences in microcirculation and macrocirculation were evaluated for two CVP groups: CVP ≤ 12 mmHg (low CVP) and CVP > 12 mmHg (high CVP). This cut-off value is the lower limit of the advised CVP goal (12–15 mmHg) for the resuscitation of mechanically ventilated septic patients according to the Surviving Sepsis Campaign (SSC) guidelines [14]. Furthermore, perfusion pressure, defined as MAP minus CVP, was calculated for each group and each time point. Backwards stepwise logistic regression was employed to detect determinants of a capillary MFI < 2.6. This threshold is the lower limit of the 95% confidence interval for healthy subjects [15]. In a recent study, this cut-off value was confirmed for the response of the microcirculation during fluid therapy, underlining the clinical significance of this threshold [16]. Predictors in univariate logistic regression with P < 0.25 for a capillary MFI were included for subsequent modeling. Tested predictors were perfusion pressure, lactate level, norepinephrine and dopamine dose, PEEP, cardiac index, SvO_2_, CVP as a continuous variable, CVP > 12 mmHg and MAP.

Evolution over time (i.e. between T0 and T30) was also evaluated for both microcirculation and macrocirculation.

The Kolmogorov-Smirnov test was used to test whether data were distributed normally; a Student’s T-test was used to test for differences between groups. Data are presented as mean ± standard deviation unless specified otherwise. A P <0.05 was considered statistically significant.

## Results

Seventy patients with an APACHE II score of 21 ± 6.5 were included; ICU mortality was 21%. Table 1 presents a summary of baseline characteristics.

**Table 1:** Baseline patient characteristics

	**Patients, baseline (n = 70 patients)**
**Age (years)**	62 ± 16
**Male**	43 (61)
**APACHE II**	21 ± 6.5
**SOFA**	10 ± 3
**Mechanical ventilation**	69 (99)
**Sepsis source**	
***Lung***	24 (34)
***Abdomen***	31 (44)
***Urinary tract***	4 (6)
***Other***	11 (16)

Mean ± SD or n (%).

### Static measurements

**Figure 1:**
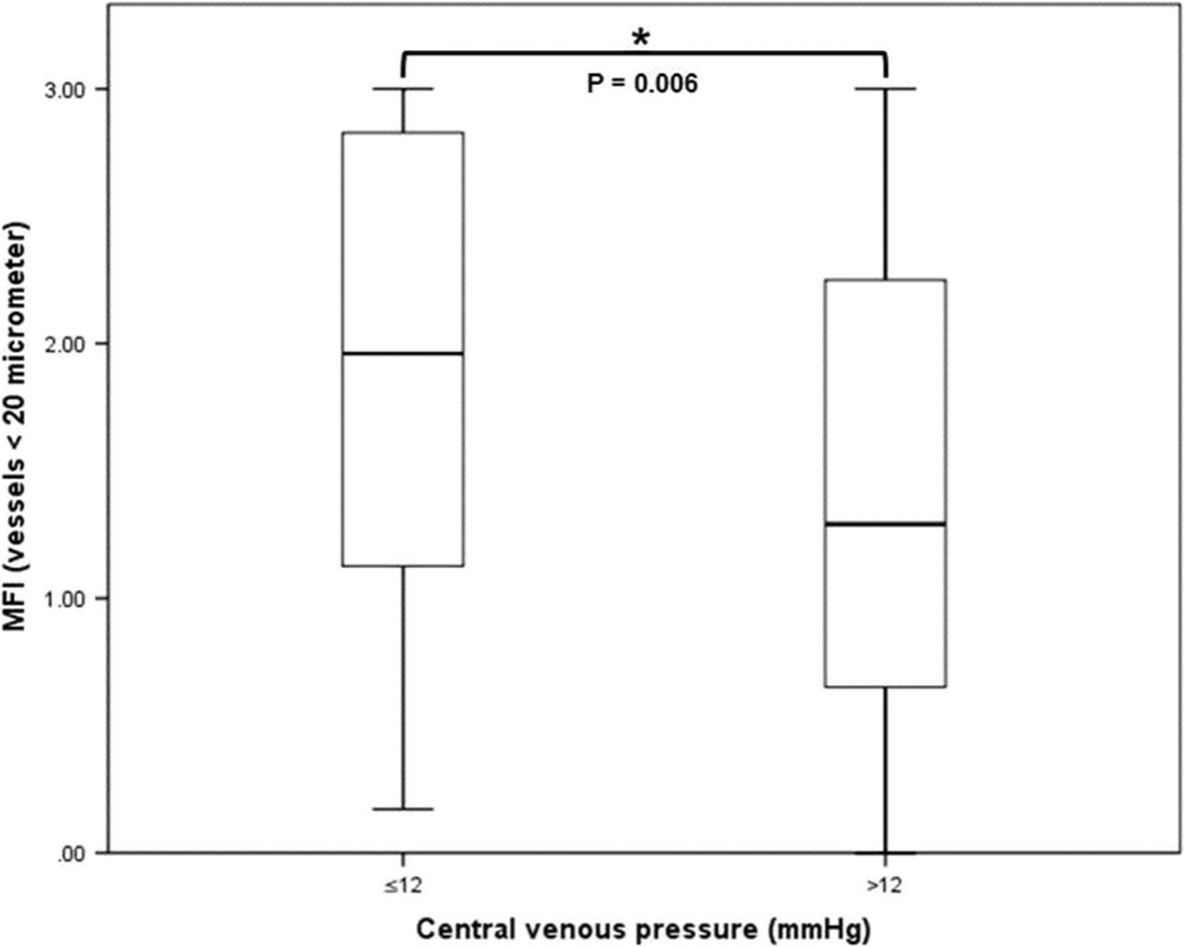
**Boxplots of microvascular flow index (MFI) in patients with a central venous pressure (CVP) ≤ or > 12 mmHg.** *For differences in MFI between CVP groups. A P < 0.05 was considered statistically significant.

Out of the 140 combined measurements of CVP and microcirculation in the first hour of completed resuscitation, 80 measurements (57%) were obtained in patients with a CVP ≤ 12 mmHg. CVP in the ‘high’ CVP group (CVP > 12 mmHg) was significantly higher in comparison to the low CVP group (16.6 ± 2.4 vs. 9.0 ± 2.8 mmHg, P =0.000). MFI and PPV were significantly lower in patients with a ‘high’ CVP (1.44 ± 0.94 vs. 1.89 ±0.91, P = 0.006; and 88 ± 21 vs. 95 ± 8%, P = 0.006) (Figure 1). In the ‘low’ CVP group, 66% of patients had a capillary MFI < 2.6, whereas 83% of patients with a CVP > 12 mmHg had a MFI < 2.6 mmHg (P = 0.023). PVD and TVD did not differ significantly between both CVP groups. At the macrohemodynamic level, both CI, MAP and perfusion pressure did not differ significantly between groups. However, SvO_2_ was significantly higher in patients in the ‘low’ CVP group, and lactate levels significantly lower. A non-significant difference in perfusion pressure was observed (Table 2). In a multivariate logistic regression analysis, the only significant predictor for an abnormal MFI was a CVP > 12 mmHg (Odds ratio 2.5 (95% confidence interval 1.1-5.8), P = 0.026).

**Table 2:** Haemodynamic variables and microcirculatory parameters for all measurements as well as per CVP group

	**All (n = 140)**	**CVP ≤12 mmHg (n = 80)**	**CVP >12 mmHg (n = 60)**	**P **^**a**^
**MAP (mmHg)**	70 ± 13	68 ± 12	75 ± 13	0.443
**Cardiac index (l/min/m^2^)**	4.2 ± 1.3	4.3 ± 1.1	4.0 ± 1.5	0.121
**CVP (mmHg)**	12.3 ± 4.6	9.0 ± 2.8	16.6 ± 2.4	0.000*
**Perfusion pressure (mmHg)**	57 ± 13	55 ± 13	59 ± 12	0.055
**PEEP (cm H_2_O)**	13 ± 3	13 ± 3	14 ± 3	0.053
**SvO_2_ (%)**	71 ± 7.9	72 ± 7.0	68 ± 8.5	0.003*
**Lactate (mmol/L)**	2.6 ± 2.2	1.8 ± 1.4	3.6 ± 2.6	0.000*
**Dopamine (n,%, μg/kg/min)**	121, 86, 6.7 ± 3.1	66, 83, 5.9 ± 2.7	55, 92, 7.9 ± 3.1	0.000*^,b^
**Norepinephrine (n,%, μg/kg/min)**	68, 49, 0.14 ± 0.15	32, 40, 0.10 ± 0.06	36, 60, 0.18 ± 0.19	0.013*^,b^
**MFI (AU)**	1.70 ± 0.95	1.89 ± 0.91	1.44 ± 0.94	0.006*
**PPV (%)**	92 ± 15	95 ± 8	88 ± 21	0.006*
**TVD (mm/mm^2^)**	14.18 ± 2.11	14.31 ± 1.95	14.00 ± 2.31	0.410
**PVD (mm/mm^2^)**	13.70 ± 7.88	14.66 ± 9.81	12.42 ± 3.8	0.066

Mean ± SD unless specified otherwise. CVP = Central venous pressure. MAP = mean arterial pressure. Perfusion pressure = MAP minus CVP. SvO_2_ = mixed venous oxygen saturation. MFI = microvascular flow index. AU = arbitrary units. TVD = total vessel density. PPV = percentage of perfused vessels. PVD = perfused vessel density. TVD, MFI, PPV and PVD for small vessels, i.e. < 20 μmeter. *P < 0.05 is considered statistically significant. ^a^ For differences between CVP-groups. ^b^for difference in dose between CVP groups.

### Dynamic measurements

Table 3 provides details on changes over time in macrohemodynamic and microcirculatory variables between T0 and T30. MFI was significantly higher at T30 as compared to T0 values (1.58 ± 1.62 vs. 1.81 ± 0.92, P = 0.027). This was accompanied by a significant decrease in CVP, MAP and perfusion pressure between T0 and T30. No significant change in cardiac index was observed.

**Table 3:** Haemodynamic variables and microcirculatory parameters for T0 and T30

	**T = 0 (n = 70 patients)**	**T = 30 (n = 70 patients)**	**P **^**a**^
**MAP (mmHg)**	71 ± 12	68 ± 13	0.008*
**Cardiac index (l/min/m^2^)**	4.2 ± 1.3	4.1 ± 1.3	0.291
**CVP (mmHg)**	12.6 ± 4.8	11.9 ± 4.3	0.017*
**Perfusion pressure (mmHg)**	59 ± 13	56 ± 13	0.034*
**PEEP (cm H_2_O)**	13 ± 3	13 ± 3	0.425
**SvO_2_ (%)**	71 ± 7.4	70 ± 8.4	0.141
**Lactate (mmol/L)**	2.6 ± 2.2	2.6 ± 2.3	0.779
**Dopamine (n,%, μg/kg/min)**	60, 86, 5.8 ± 3.7	61, 87, 5.9 ± 3.7	0.772
**Norepinephrine (n,%, μg/kg/min)**	33, 47, 0.07 ± 0.11	35, 50, 0.07 ± 0.15	0.401
**MFI (AU)**	1.58 ± 1.62	1.81 ± 0.92	0.027*
**PPV (%)**	91 ± 18	94 ± 12	0.197
**TVD (mm/mm^2^)**	14.27 ± 2.22	14.08 ± 2.01	0.415
**PVD (mm/mm^2^)**	14.11 ± 10.84	13.28 ± 2.71	0.496

Mean ± SD unless specified otherwise. T0: directly after fulfillment of resuscitation endpoints. T30: 30 minutes after T0. CVP = Central venous pressure. MAP = mean arterial pressure. Perfusion pressure = MAP minus CVP. PEEP = positive end-expiratory pressure. SvO_2_ = mixed venous oxygen saturation. MFI = microvascular flow index. AU = arbitrary units. TVD = total vessel density. PPV = percentage of perfused vessels. PVD = perfused vessel density. TVD, MFI, PPV and PVD for small vessels, i.e. < 20 meter. ^a^For differences between T0 and T30. *P < 0.05 is considered statistically significant.

## Discussion

Our results show an association between elevated CVP and impairment of microcirculatory blood flow in the early phase of human sepsis. At the same time, MAP and perfusion pressure did not differ significantly between both CVP groups. Moreover, in the short time frame of our analysis, we observed a significant rise in MFI in combination with a reduction in CVP, despite a decrease in MAP and perfusion pressure. These observations are compatible with our hypothesis that the ‘classical’ perfusion pressure, defined as MAP minus CVP, may not reflect the true driving pressure over the microcirculation. In this respect, two factors should be taken into consideration: 1) inflow pressure of the microcirculation may significantly differ from MAP as a result of post-arteriolar pressure drop and 2) the microcirculation may be considered as a low pressure compartment, with hydrostatic pressures slightly above CVP. However, diffusion distance seems to be unaffected, as reflected by the absence of a significant difference in TVD and PVD between CVP groups as well as over time. This may be explained by the fact that upregulation of the number of perfused capillaries, to compensate for a reduction in convective oxygen transport, may occur outside the timeframe of our observations [17].

Data on the effect of elevated venous pressure on microcirculatory blood flow are limited: SDF imaging of renal blood flow in pigs showed a decrease in renal MFI in intra-abdominal hypertension, which is a model for venous outflow obstruction [18]. In an experimental setting, aiming for a CVP < 10 mmHg by intravenous administration of nitroglycerin in gastric tube reconstruction in pigs resulted in a higher microvascular blood flow as measured by laser Doppler flowmetry in comparison to controls, without being influenced by increases in MAP [19]. The same group reported an increase in microvascular blood flow after topical administration of nitroglycerin in gastric tube reconstruction in humans, being compatible with the hypothesis that venous outflow obstruction results in impairment of microvascular flow [20]. Several studies report impairment of microvascular perfusion in small increases in venous pressure by venous congestion plethysmography in humans [21,22].

It is conceivable that raising CVP under specific circumstances may be beneficial to tissue perfusion [16]. However, several studies point towards an ambiguous role of CVP in resuscitation of critically ill patients. Not only CVP failed as a useful measure for the assessment of preload and fluid responsiveness [23], a CVP > 12 mmHg was also associated with a higher mortality in this specific patient group in the early phase of resuscitation [24]. Our data add to the understanding that taking elevated CVP levels as a general endpoint will not automatically result in improved organ perfusion. Therefore, it is conceivable that CVP guided resuscitation as advocated by for instance Surviving Sepsis Campaign (SSC) guidelines might have an undesirable effect on microcirculatory perfusion [14].

Our study has several limitations. Due to the post hoc design of the study, we were limited in the ability to explore the complex relationship between venous pressure and microcirculatory blood flow. Therefore, it is of utmost importance to stress that our finding is merely hypothesis generating. Imbalances between the two CVP groups in inotrope use, lactate levels and SvO_2_ may both be explained as confounders, exaggerating the observed differences in microcirculatory perfusion. Extravascular pressures such as intra-abdominal pressure and positive end expiratory pressure (PEEP) settings may also have influenced CVP. PEEP level did not differ significantly between groups, nor did it change over time. In this study, data on intra-abdominal pressure are lacking. However, correction for these potential confounders in multivariate analysis did not eliminate the observed differences in microvascular perfusion between CVP groups. Moreover, the potential confounding factors may also serve as additional markers of impaired organ perfusion, underlining the importance of the observed differences between the groups.

SDF imaging of the sublingual microcirculation was performed, but venous pressure was not measured at this level. It is imaginable that venous pressure in the superior vena cava is not representative for venous pressure at the sublingual site and that this might have influenced the association between venous pressure and the sublingual microcirculation.

We are also aware of the fact that microcirculatory blood flow is determined by other factors than inflow and outflow pressures alone. Not only may there be a further pressure drop within the capillary and venular compartment, it is also of note that microcirculatory flow regulation is not a static process. In reality, vasomotion is the constant opening and closing of capillaries under influence of downstream hypoxic signals [25]. However, in the clinical setting, this complexity of microcirculatory flow is a complete black box. In previous papers, authors have tried to establish a clear relationship between the input signal (i.e. arterial pressure) and the microcirculation and were unable to do so [1].

Our data were limited to a short time frame in the early course of sepsis resuscitation. Therefore, extrapolation of our findings to a larger time window is difficult. It was decided to limit the evaluation of SDF and CVP data to the measurements obtained at 0 and 30 minutes, because we observed a tendency towards progression of MFI towards 3 in the majority of patients during the 24 hour study period in the original data set [9]. Due to this regression to the mean phenomenon, it was expected that the a priori probability for detecting an association between CVP and microcirculation was highest in the early phase after initial sepsis resuscitation.

## Conclusion

The observed association between elevated CVP and impairment of microcirculatory perfusion is in line with physiological theory that microcirculatory perfusion pressure is predominantly determined by CVP as an outflow obstruction. This may have especially potential consequences for the use of a elevated CVP levels as a resuscitation endpoint. Clearly, our study was intended merely to explore the possibility of an association between microcirculatory parameters and elevated CVP in sepsis. The study design does not allow for making assumptions regarding causality. Further research is needed to clarify the role of elevated CVP in microvascular dysfunction in sepsis.

### Key messages

Microcirculatory perfusion pressure can be defined as post-arteriolar pressure minus venular pressure and is much closer to venous pressure as to arterial pressure.

An increased venous pressure is associated with impairment of microcirculatory perfusion in patients with severe sepsis and septic shock.

This observation needs to be elucidated further in terms of a causal relationship, but could have potential consequences for resuscitation guidelines that are based on the assumption that elevated CVP will automatically improve organ perfusion.

## Abbreviations

APACHE: Acute physiology and chronic health evaluation; CVP: Central venous pressure; CI: Cardiac index; ICU: Intensive care unit; MAP: Mean arterial pressure; MFI: Microvascular flow index; PEEP: Positive end-expiratory pressure; PPV: Percentage of perfused vessels; PVD: Perfused vessel density; SDF: Sidestream dark field; SOFA: Sequential organ failure assessment; SSC: Surviving sepsis campaign; SvO2: Mixed venous oxygen saturation; TVD: Total vessel density.

## Competing interests

C. Ince is the inventor of SDF technology, which is commercialized by MicroVision Medical. He has been a consultant for this company in the past, but he has broken all contact with this company for more than four years now, and he has no competing interests other than his commitment to promote the importance of the microcirculation in the care of critically ill patients.

## Authors’ contributions

NV contributed to the study design, performed statistical analysis and wrote the manuscript draft. CI contributed to the study design and revised the manuscript critically. ECB was involved in data collection, SDF imaging and analysis, study design and writing the manuscript. All authors read and approved the final manuscript.

## Pre-publication history

The pre-publication history for this paper can be accessed here:

http://www.biomedcentral.com/1471-2253/13/17/prepub
